# Delayed Puberty Due to a WDR11 Truncation at Its N-Terminal Domain Leading to a Mild Form of Ciliopathy Presenting With Dissociated Central Hypogonadism: Case Report

**DOI:** 10.3389/fped.2022.887658

**Published:** 2022-06-03

**Authors:** Sebastián Castro, Franco G. Brunello, Gabriela Sansó, Paula Scaglia, María Esnaola Azcoiti, Agustín Izquierdo, Florencia Villegas, Ignacio Bergadá, María Gabriela Ropelato, Marcelo A. Martí, Rodolfo A. Rey, Romina P. Grinspon

**Affiliations:** ^1^Centro de Investigaciones Endocrinológicas “Dr. César Bergadá” (CEDIE), CONICET – FEI – División de Endocrinología, Hospital de Niños Ricardo Gutiérrez, Buenos Aires, Argentina; ^2^Departamento de Química Biológica, Instituto de Química Biológica de la Facultad de Ciencias Exactas y Naturales (IQUIBICEN) CONICET, Ciudad Universitaria, Buenos Aires, Argentina; ^3^Unidad de Medicina Traslacional, Hospital de Niños Ricardo Gutiérrez, Buenos Aires, Argentina; ^4^Servicio de Genética, Hospital de Niños Ricardo Gutiérrez, Buenos Aires, Argentina; ^5^Departamento de Biología Celular, Histología, Facultad de Medicina, Universidad de Buenos Aires, Embriología y Genética, Buenos Aires, Argentina

**Keywords:** AMH, GnRH, hyposmia, Kallmann syndrome, puberty, testosterone

## Abstract

Pubertal delay in males is frequently due to constitutional delay of growth and puberty, but pathologic hypogonadism should be considered. After general illnesses and primary testicular failure are ruled out, the main differential diagnosis is central (or hypogonadotropic) hypogonadism, resulting from a defective function of the gonadotropin-releasing hormone (GnRH)/gonadotropin axis. Ciliopathies arising from defects in non-motile cilia are responsible for developmental disorders affecting the sense organs and the reproductive system. WDR11-mediated signaling in non-motile cilia is critical for fetal development of GnRH neurons. Only missense variants of *WDR11* have been reported to date in patients with central hypogonadism, suggesting that nonsense variants could lead to more complex phenotypes. We report the case of a male patient presenting with delayed puberty due to Kallmann syndrome (central hypogonadism associated with hyposmia) in whom the next-generation sequencing analysis identified a novel heterozygous base duplication, leading to a frameshift and a stop codon in the N-terminal region of WDR11. The variant was predicted to undergo nonsense-mediated decay and classified as probably pathogenic following the American College of Medical Genetics and Genomics (ACMG) criteria. This is the first report of a variant in the WDR11 N-terminal region predicted to lead to complete expression loss that, contrary to expectations, led to a mild form of ciliopathy resulting in isolated Kallmann syndrome.

## Introduction

Pubertal delay in males is frequently due to constitutional delay of growth and puberty, but pathologic hypogonadism should be considered (1–3). A delay of puberty is diagnosed when no clinical signs of pubertal onset are present by the age of 14 (4). In approximately 2/3 of the cases, this is due to a normal developmental variant called constitutional delay of growth and puberty (5), but this is an exclusion diagnosis and causes of pathologic hypogonadism should be explored. After ruling out general chronic or acute illnesses and primary testicular failure, the main differential diagnosis is central (or hypogonadotropic) hypogonadism, characterized by low testosterone secretion by the testes due to an impaired gonadotropin-releasing hormone (GnRH)/gonadotropin axis function (3, 4, 6–8). The GnRH neurons originate from the olfactory placode during the first trimester in the human fetus and migrate to the hypothalamus, following the axon guidance of the vomeronasal nerve. Mutations in genes involved in GnRH neuron and olfactory nerve development result in Kallmann syndrome, where congenital central hypogonadism is associated with hyposmia or anosmia (4, 7, 8).

Motile cilia confer cell motility, e.g., respiratory cilia and sperm flagellum, whereas non-motile primary cilia have an essential structural role in intracellular signal transduction. Ciliopathies arising from defects in non-motile cilia underlie the pathogenesis of developmental disorders affecting the sense organs and the reproductive, cardiocirculatory, and central nervous systems (9). The primary cilia play a central role in WDR11-mediated Hedgehog signaling that is critical for embryonic patterning of GnRH neurons and the olfactory bulb. WDR11 belongs to the WD (tryptophan-aspartate) repeat-protein family (10), encoded by a 58-kb gene containing 29 exons at 10q26.12 (OMIM *606417). Monoallelic missense variants in the WD domains located in the central region of the protein have been reported in 7 cases with an autosomal dominant form of central hypogonadism probably resulting from WDR11 haploinsufficiency or a dominant negative effect (11). No truncating mutations have been described hitherto (12), suggesting that nonsense or frameshift variants in the N-terminal domain might lead to more severe complex phenotypes, as observed in the *WDR11*-null mice (10). Here, we describe the first case of a 21-year-old male patient presenting with delayed puberty due to Kallmann syndrome, carrying a variant in *WDR11* ostensibly leading to a truncated protein that resulted, contrary to expectations, in a mild form of ciliopathy.

## Materials and Methods

### Clinical Assessment and Hormone Assays

The height was measured using a wall-mounted stadiometer, and the weight was determined with a calibrated scale, and both were expressed as SD score (SDS) based on the Argentine population reference (13). The pubertal stage was assessed according to Marshall and Tanner (14). The penile size was compared to the standardized data of the Argentine population (15). The bone age was estimated using the Greulich and Pyle’s radiographic atlas of skeletal development (16).

Serum follicle-stimulating hormone (FSH), luteinizing hormone (LH), testosterone, and anti-Müllerian hormone (AMH) were measured using validated assays, as previously published (17, 18). A GnRH iv infusion test (100 μg GnRH, Luteoliberina; Elea SACIFyA; 0.83 μg/min for 120 min) was performed by using the AVI 270 infusion pump (AVI Inc. 3M Healthcare), as previously published (19). LH and FSH were determined by immunofluorometric assays in serum at 0, 15, 30, 45, 60, and 120 min. Total insulin-like growth factor 1 (IGF1), and thyroxine (T4), free T4, thyrotropin (TSH), cortisol, dehydroepiandrosterone sulfate (DHEA-S), adrenocorticotropin (ACTH), and prolactin were determined as previously reported (20, 21).

### Next-Generation Sequencing and Filtering

The genomic DNA was extracted from peripheral venous blood cells using the Gentra Puregene Blood Kit (Qiagen) (22). The DNA was quantified using a high-performance microvolume spectrophotometer Nanophotometer^®^ NP60 (Implen Inc.), and the DNA concentration was normalized to 10 ng/μl using a fluorometer Qubit^®^ 3.0 (Invitrogen). The DNA purity was assessed by measuring the absorbance ratio 260/280 nm: further DNA sample processing was performed only if the ratio was between 1.8 and 2.1. DNA library preparation and exon capture from the proband were performed using the TruSight One^®^ sequencing panel (Illumina), which provides coverage of 4,813 genes associated with known Mendelian genetic disorders (∼12 Mb genomic content^1^). The quality of genomic DNA fragmentation was controlled using a capillary system Fragment Analyzer™ (Advanced Analytical). Next-Generation Sequencing (NGS) by synthesis with fluorescent reversible terminator deoxyribonucleotides (23) was performed using a NextSeq 500^®^ system (Illumina) at the Translational Medicine Unit of the Buenos Aires Children’s Hospital (Unidad de Medicina Traslacional, Hospital de Niños Ricardo Gutiérrez, Buenos Aires).

We used the strategy recommended by the Broad Institute in the Genome Analysis Toolkit (GATK best practices™) for preprocessing, variant calling, and refinement. The raw sequence data were mapped to the 1000-Genomes phase II reference genome (GRCh37 version hs37d5) using the BWA-MEM algorithm of the Burrows-Wheeler Aligner software (24) and visualized with the Integrative Genomics Viewer (IGV v.1.4.2), Broad Institute of Massachusetts Institute of Technology and Harvard, Cambridge, Massachusetts, United States (25). Duplicates were removed using Picard (Broad Institute).

Variant filtering and prioritization were performed on B_platform,^2^ as previously reported using inhouse pipelines (26). The variant call format (VCF) file was annotated with ClinVar,^3^ gnomAD,^4^ and dbSNP^5^ databases. Candidate variants were selected when minor allele frequency (MAF) was < 1% in gnomAD exomes and genomes in the 1000 Genomes and in Bitgenia’s database of over 100 Argentine control individuals.^6^ For further analysis, single-nucleotide variants (SNVs) and indels with a read depth ≥ 10× and a Phred quality score ≥ 20, and Genotype Quality (GQ) score ≥ 60 among the 42 candidate genes for central hypogonadism available in the TruSight One panel were considered. The list included *ANOS1 (KAL1), AMH, AXL, CHD7, DAX1, DCC, FGF8, FGFR1, FSHB, GHSR, GLI3, GNRH1, GNRHR, HESX1, HS6ST1, IGFALS, KISS1, KISS1R, KLB, LEP, LEPR, LHB, LHX4, MKRN3, MSX1, NR0B1 (DAX1), NSMF, OTX2, PCSK1, PNPLA6, POLR3A, POLR3B, PROK2, PROKR2, PROP1, SEMA3A, SEMA3E, SOX2, SOX10, TAC3, TACR3*, and *WDR11*.

Various pathogenicity predictors, such as CADD,^7^ Mutation Taster,^8^ Polyphen2,^9^ REVEL,^10^ and SIFT,^11^ were used to predict variant implications on protein function. Finally, we classified the variants according to their potential pathogenicity using the American College of Medical Genetics and Genomics (ACMG) guidelines for variant interpretation (27). The following references sequences were used: GRCh37 (Human genome), WDR11: NG_023290.1 (gene), NM_018117.12 (mRNA), NP_060587.8 (protein).

### Sanger Sequencing

Relevant variants identified in the proband were confirmed by Sanger sequencing of genomic DNA from the proband and his parents. *WDR11* exon 2 was amplified by PCR with GoTaq^®^ DNA Polymerase (Promega) and the following primers: forward 5′-AGTCGTCCTGCTTTGTTCTGT-3′ and reverse 5′-ACATGTTAGCGTCAAAGTGGGA-3′. The products were sequenced using an ABI 3500 Genetic Analyzer (Applied Biosystems) at the Translational Medicine Unit of the Buenos Aires Children’s Hospital.

## Case Report

### Clinical Observation

**FIGURE 1:**
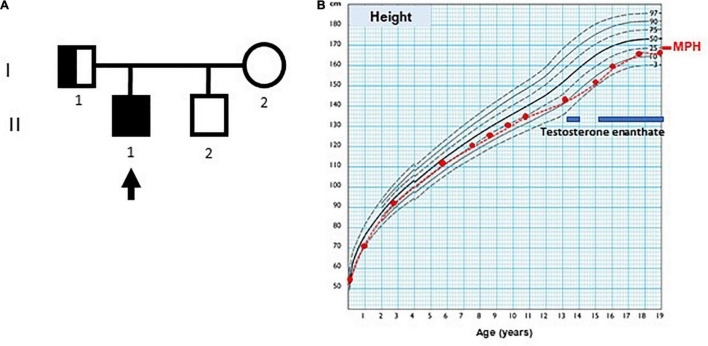
Case pedigree and growth chart. **(A)** Pedigree: the arrow indicates index case; circle, females; square, males; full black square, complete phenotype (small genitalia and hyposmia); half black square, incomplete phenotype (normal genitalia development and olfactory dysfunction). **(B)** Growth chart of index case, using Argentine standards for males. MPH: adjusted mid-parental height. The blue bars indicate periods of testosterone enanthate treatment.

The index case (Figure 1, individual II.1) was the first of two brothers, born to a healthy mother (individual I.2) and a father with hyposmia (individual I.1), as the only remarkable family background. His mother was 14 years old by the time of her menarche and her height was 159.7 cm. His father’s height was 164 cm, and the adjusted mid-parental height was 168.1 cm (30th centile). His brother (individual II.2), who was 5 years younger than the proband, was a healthy boy with normal genitalia.

At the age of 3 months, the index case was referred to our hospital because of micropenis: penile length was 1 cm (−6.4 SDS) and width 0.7 cm (−3.2 SDS). His body length and weight were normal as well as his neurological development. The testicular volume was 1 ml bilaterally, i.e., the lowest limit of normal for age according to Prader’s orchidometer (28). The assessment of the gonadal axis at the time of postnatal physiological activation revealed low serum concentrations of LH and testosterone, AMH in the lower limit of the normal range, and normal plasma concentration of FSH (Table 1). Central hypogonadism was suspected.

**TABLE 1:** Laboratory findings in the index case at different ages.

	3 months	Ref.	9 years	Ref.	13 years 2 months	Ref.
LH (IU/l)	0.05	0.50–5.00	0.58	0.10–2.80	0.34	0.10–2.80
FSH (IU/l)	0.60	0.40–2.50	2.34	0.50–2.50	1.37	0.50–2.50
Testosterone (ng/dl)	14	100–300	<10	10–100	<10	10–100
AMH (pmol/l)	237	250–680	430	250–1,300	444	250–1,300
TSH (mIU/l)					2.77	0.50–6.50
Free T4 (ng/dl)					1.05	0.80–2.00
ACTH (pg/ml)					32	<48
Cortisol (μg/dl)					13	6–21
DHEA-S (μg/l)					1,010	600–2,880
Prolactin (ng/ml)					13	3–25
IGF1 (ng/ml)					420	61–373
IGFBP3 (μg/ml)					4.7	2.2–5.6

*Ref: reference ranges for age and Tanner G1 stage (18).*

His growth and development were normal during childhood (Figure 1), but his genitalia remained small: at the age of 9 years his penis length was 3 cm (−2.3 SDS). The testicular volume was normal, 2 ml bilaterally. While the serum concentrations of LH and testosterone remained at the low levels expected for age during childhood, the baseline serum concentrations of FSH and AMH caught up to the normal reference values (Table 1).

By age 13 years 2 months, he was still prepubertal (Tanner stage G1), with sparse pubic hair (PH2), and testicular volume 3 ml bilaterally. Bone age was 13 years old, height was 144 cm (17th centile), and weight was 44 kg (25th centile). He referred mild hyposmia and psychosocial distress related to his pubertal delay as compared to his peers. An MRI scan revealed a bilateral reduction in the size of the olfactory bulbs, with olfactory sulci depth of 7 mm on both sides, leading to the clinical suspicion of Kallmann syndrome. A treatment with testosterone enanthate 50 mg intramuscular (IM) per month was given for 6 months to promote the development of secondary sex characteristics and growth spurt.

When he was 15 years old and off testosterone, his height and growth velocity were impaired (Figure 1B). His physical appearance was still prepubertal (G1, testicular volume 3 ml bilaterally) with pubic hair development (PH3) due to testosterone treatment and probably also to the occurrence of adrenarche, as reflected in serum levels of DHEA-S (Table 1). A GnRH infusion test showed an insufficient serum LH peak, with an adequate FSH peak according to validated reference levels in our laboratory (19) (Table 2). The basal testosterone was low, and AMH was in the normal range for G1 stage. Final clinical diagnosis was Kallmann syndrome (partial central hypogonadism, with a dissociated axis function showing a more affected LH-Leydig cell than FSH-Sertoli cells axis), and he was started again on testosterone enanthate 100 mg per month, with progressive increases up to 250 mg. At the age of 20 years, his adult height was 164 cm, at −0.6 SDS with respect to the mid-parental height. His penile length was 5.5 cm (−0.6 SDS) and width was 2 cm (−1.3 SDS), with testicular volumes 4 ml bilaterally, 6 months off testosterone undecanoate treatment (1,000 mg IM every 3 months). Serum LH was 0.25 IU/l, FSH 0.89 IU/l, testosterone 133 ng/dl, and AMH 301 pmol/l. He was socially adapted and studying at medical school.

**TABLE 2:** Result of a GnRH infusion test in the index case.

	Basal	15 min	30 min	45 min	60 min	120 min	Ref. cut-off
LH (IU/l)	0.65	3.86	5.56	4.19	3.96	3.28	5.80
FSH (IU/l)	1.73	3.23	4.99	6.29	6.42	6.90	4.60

*Ref. cut-off: reference cut-off value for the diagnosis of central hypogonadism (19).*

### Identification of a Genetic Candidate Variant for Central Hypogonadism

**FIGURE 2:**
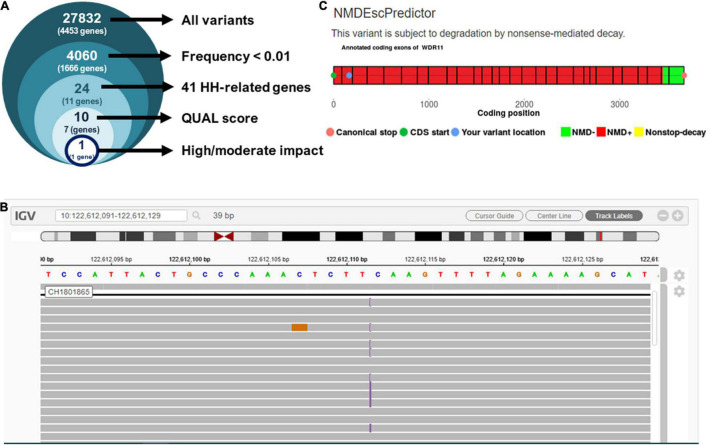
Analysis of next-generation sequencing results. **(A)** Prioritization of variants found in the proband by targeted exome NGS. **(B)** Integrative Genome Viewer (IGV) visualization of variant NM_018117.12:c.163dup, p.(Gln55Pro fs7*). **(C)** Prediction of nonsense-mediated decay using NMDEscPredictor.

The targeted exome NGS in the proband showed an average coverage of 45× and > 99% of bases in the target autosomal genes with ≥10× coverage and ≥ 20 QUAL score. The initial analysis identified 27,832 variants in 4,453 genes. Filtering for candidate variants with MAF < 1% in gnomAD, the 1,000 Genomes and Bitgenia’s Argentine database yielded 4,060 variants in 1,666 genes. Further analysis of SNVs and indels with a read depth ≥ 10×, a Phred quality score ≥ 20, and GQ score ≥ 60 among the 41 candidate genes for central hypogonadism available in the TrueSight One sequencing panel selected one variant (Figure 2A) at chromosome position 10.122612112, corresponding to exon 2 of *WDR11* (Figure 2B). The variant was NM_018117.12*(WDR11)*:c.163dup, p.(Gln55Pro fs7*), indicating a change from glutamine to proline at position 55 of the protein, with a change in the reading frame leading to a stop codon 7 amino acids downstream. The position was read with a depth of 56×, with the cytosine duplication at position 163 in 25 reads and the reference allele in 31 reads, clearly compatible with a heterozygous presentation. Sanger sequencing confirmed the existence of the heterozygous variant in the proband’s father and its absence in his mother. The variant was not reported in any of the consulted databases, i.e., gnomAD exomes and genomes, 1,000 Genomes and Bitgenia’s database of > 100 Argentine controls. The NM_018117.12*(WDR11)*:c.163dup, p.(Gln55Pro fs7*) variant was classified as likely pathogenic according to the ACMG criteria, since it met the requirement of one strong (PS3), one moderate (PM2), and one supporting (PP1) criterion for pathogenicity, as follows: PS3 well established *in vitro* or *in vivo* functional studies supportive of a damaging effect on the gene or gene product (10, 12), PM2 variant not found in gnomAD exomes/genomes, and PP1 co-segregation with disease in proband and his father (10, 11).

### Variant Modeling

Since the sequence analysis of the variant showed that it leads to a frameshift in exon 2, after residue Gln55 with the subsequent occurrence of a stop codon, we evaluated the likelihood that it undergoes nonsense mediated decay (NMD) using the NMDEscPredictor.^12^ As expected, it predicted NMD for the present case (Figure 2).

## Discussion

In this study, we report the first case of a truncating variant of *WDR11* resulting in a mild form of ciliopathy responsible for central hypogonadism in a male. Our observation indicates that nonsense *WDR11* variants do not result in severe, complex phenotypes in humans, as could have been expected from observations in mice (10) or from larger deletions encompassing the *WDR11* locus such as the 10q26 deletion syndrome in humans (29).

The cilia consist of a membrane lipid bilayer that surrounds a microtubule-based axoneme, assembled from the centrosome (9). In male reproductive physiology, the formation of the flagellum during spermiogenesis is one of the paradigmatic ciliogenic processes. Like other motile cilia, the sperm flagellum functions as a propulsive organelle enabling the male gamete to reach the ovum during the process of fertilization. Conversely, primary, non-motile cilia are involved in cell signaling after the ciliary membrane receptors are excited by physical stimuli, hormones, chemokines, growth factors, or morphogens. Ciliopathies may result from defects in motile or non-motile cilia (9). Genetic disorders of respiratory motile cilia and sperm flagella, leading to primary ciliary dyskinesia or Kartagener’s syndrome, is a typical example of the former. On the other hand, polycystic kidney disease; nephronophthisis; and Bardet-Biedl, Joubert, and Meckel syndromes represent examples of non-motile ciliopathies. Hedgehog signaling plays a critical role in ciliary function implicated in embryonic patterning, and WDR11 has specifically been shown to be essential in GnRH neuron physiology. Elegant experimental studies in mice have clearly demonstrated that, following hedgehog stimulus, WDR11 shuttles from the cilium to the nucleus, regulates GLI3 proteolytic processing, and cooperates with EMX1 in transcriptional activation of target gene expression leading to GnRH production (10). Therefore, the reproductive disorder described in our patient results from a ciliopathy that, rather than impairing male germ cell flagellum development, disrupts non-motile ciliary function at the hypothalamic GnRH neuron level, resulting in an impaired testicular hormone production ultimately leading to spermatogenic failure.

*Wdr11*-null mice show reduced numbers of GnRH neurons and a reduced expression of the LH beta subunit in males (10), thus providing experimental support to the biological plausibility for *WDR11* mutations to underlie central hypogonadism. All *WDR11* variants described to present are missense (12). The case we describe here is the first to bear a base stop codon in exon 2, with predicted mRNA NMD. Moreover, even if the mRNA was translated, it would yield a small polypeptide containing less than 2 complete WD domains, while the WRD11 structure contains 2 beta propellers formed by the 9 WD repeat domains, which are essential for its scaffold function mediating GLI3 trafficking from the ciliary base to the nucleus, resulting in GLI3/EMX1-mediated transcriptional regulation of the target genes (11).

Hypogonadism usually encompasses the functional damage of all testicular cell populations, i.e., low androgen production by Leydig cells, impaired AMH secretion by Sertoli cells, and disrupted spermatogenesis. Less frequently, only one cell population (Leydig, Sertoli, or germ cells) is primarily affected, resulting in a dissociated hypogonadism (30). Boys with failure to enter puberty due to central hypogonadism obviously show a deficiency in the LH-Leydig cell axis, while the FSH-Sertoli cell axis is usually overlooked (31, 32). The existence of microorchidism prompts the suspicion of concomitant FSH insufficiency, since this gonadotropin is critical for Sertoli cell proliferation, and Sertoli cells are the main component of the testes before pubertal onset (31). In our patient, the signs of hypoandrogenism, such as micropenis and cryptorchidism, were not associated with microorchidism, and FSH and AMH levels were normal, indicating the occurrence of a dissociated hypogonadism. Dissociated central hypogonadism, with low LH and normal FSH, has been described in individuals with *TACR3* pathogenic variants (33). Interestingly, *Wdr11*-null mice have decreased LHβ-subunit but normal FSHβ-subunit expression (10), suggesting the existence of a dissociated hypogonadism, which could explain the observation in our patient.

In conclusion, a nonsense heterozygous variant in the second exon of *WDR11* results in a ciliopathy leading to a partial form of central hypogonadism, with a more affected LH and testosterone production and a relatively more preserved FSH levels, associated with hyposmia due to olfactory nerve defects (Kallmann syndrome). The deleterious effect of the *WDR11* variant on the hypothalamic-pituitary-testicular axis, resulting in pubertal delay, seems to reflect a double hit produced by an impaired signaling at the level of both GnRH neuron migration and the regulation of LHβ subunit expression in the gonadotrope. However, it does not result in a more complex phenotype affecting other organs, as could be predicted.

## Data Availability Statement

The datasets for this article are not publicly available due to concerns regarding participant/patient anonymity. Requests to access the datasets should be directed to the corresponding author.

## Ethics Statement

The studies involving human participants were reviewed and approved by Comité de Ética en Investigación, Hospital de Niños Ricardo Gutiérrez, Buenos Aires. Written informed consent to participate in this study was provided by the participants’ legal guardian/next of kin. Written informed consent was obtained from the individual(s), and minor(s)’ legal guardian/next of kin, for the publication of any potentially identifiable images or data included in this article.

## Author Contributions

MGR, MAM, RAR, and RPG: conceptualization. SC, FGB, GS, AI, and FV: methodology. PS, MEA, and MGR: validation. SC, FGB, MAM, RAR, and RPG: writing—original draft preparation. GS, PS, MEA, AI, IB, FV, and MGR: writing—review and editing. All authors have read and agreed to the published version of the manuscript.

## Conflict of Interest

The authors declare that the research was conducted in the absence of any commercial or financial relationships that could be construed as a potential conflict of interest.

## Publisher’s Note

All claims expressed in this article are solely those of the authors and do not necessarily represent those of their affiliated organizations, or those of the publisher, the editors and the reviewers. Any product that may be evaluated in this article, or claim that may be made by its manufacturer, is not guaranteed or endorsed by the publisher.

## Footnotes

1
https://www.illumina.com/products/by-type/clinical-research-products/trusight-one.html

2
https://www.bitgenia.com/b-platform/

3
https://www.ncbi.nlm.nih.gov/clinvar/

4
https://gnomad.broadinstitute.org/

5
https://www.ncbi.nlm.nih.gov/snp/

6
https://www.bitgenia.com/100exomas/

7
https://cadd.gs.washington.edu/

8
http://www.mutationtaster.org/

9
http://genetics.bwh.harvard.edu/pph2/

10
https://sites.google.com/site/revelgenomics/about

11
http://sift.bii.a-star.edu.sg/

12
https://nmdprediction.shinyapps.io/nmdescpredictor/
